# Potential Biomarkers for Asymptomatic Visceral Leishmaniasis among Iraq-Deployed U.S. Military Personnel

**DOI:** 10.3390/pathogens12050705

**Published:** 2023-05-12

**Authors:** Fernanda Fortes de Araujo, Ines Lakhal-Naouar, Nancy Koles, Sorana Raiciulescu, Rupal Mody, Naomi Aronson

**Affiliations:** 1 Department of Medicine, Uniformed Services University of the Health Sciences, Bethesda, MD 20814, USA; nancy.koles.ctr@usuhs.edu (N.K.); rupal.m.mody.civ@health.mil (R.M.); naomi.aronson@usuhs.edu (N.A.); 2 Henry M. Jackson Foundation for the Advancement of Military Medicine, Bethesda, MD 20817, USA; 3 Diagnostics and Countermeasures Branch, Walter Reed Army Institute of Research, Silver Spring, MD 20910, USA; ines.elakhalnaouar.civ@health.mil; 4 Department of Preventive Medicine and Biostatistics, Uniformed Services University of the Health Sciences, Bethesda, MD 20814, USA; sorana.raiciulescu@usuhs.edu; 5 Department of Medicine, William Beaumont Army Medical Center, El Paso, TX 79916, USA

**Keywords:** visceral leishmaniasis, asymptomatic, biomarkers

## Abstract

Visceral leishmaniasis (VL) is a chronic infection caused by *Leishmania* (*L*.) *donovani* or *L. infantum* parasites. Despite having the infection, most individuals never develop the clinical disease and are able to control the parasite and remain asymptomatic. However, some progress to symptomatic VL, leading to death if untreated. The host immune response has a major role in determining the progression and severity of the clinical manifestations in VL; several immune biomarkers of symptomatic VL have been described with interferon-gamma release as a surrogate biomarker of host cellular immunity. However, new biomarkers to identify asymptomatic VL (AVL) are needed for the identification of people at risk for VL activation. In our study, levels of chemokine/cytokine in the supernatants of peripheral mononuclear blood cells (PBMC) from 35 AVL^+^ Iraq-deployed participants, stimulated in vitro with soluble *Leishmania* antigen for 72 h, were assessed by a bead-based assay that allows the measurement of multiple analytes. PBMC of AVL-negative military beneficiaries were used as controls. Monocyte Chemoattractant Protein-1, Monokine Induced by Gamma Interferon and Interleukin-8, were detected at high levels in AVL^+^ stimulated cultures from Iraq deployers compared to uninfected controls. Measurement of chemokine/cytokine levels can identify cellular immune responses in AVL^+^ asymptomatic individuals.

## 1. Introduction

Visceral leishmaniasis (VL) is a chronic infectious disease caused by *Leishmania* (*L*.) *donovani* or *L. infantum* (*syn. L. chagasi*) intracellular protozoan parasites. Worldwide, an estimated 50,000 to 90,000 new cases of VL occur annually, and VL remains one of the major parasitic diseases with significant outbreak and mortality potential [1]. Despite being infected, most individuals never develop the clinical disease and are able to control the infection, remaining asymptomatic [2,3]. However, some people progress to the more severe VL form, which is potentially fatal if not promptly treated.

The host immune response to *Leishmania* is critical for parasite clearance or disease development [4], as it plays a major role in determining the progression and severity of the clinical manifestations in VL. Several immunological markers of symptomatic VL have been described [5,6,7,8,9,10,11]. The cellular response leading to the control of *Leishmania* infection is associated with a predominant Type 1 immune profile characterized by interferon-γ (IFN-γ), tumor necrosis factor-α (TNF-α), and interleukin (IL)-2 secretion [12,13]. Alternatively, when the parasite is able to evade host-specific immune responses, VL develops [14]. In VL, parasite infiltration of the spleen, liver, lymph nodes, and bone marrow occurs. The patient with active VL has immune responses associated with cellular anergy or a Type 2 response and production of IL-4, IL-5, IL-10, IL-13, and transforming growth factor-β (TGF-β), which do not control the infection [15].

Recent studies in VL have shown that some chemokines (IL-8/CXCL8, MCP-1/CCL2, MIG/CXCL9, and IP-10/CXCL10) are associated with a Th1 cellular response [15]. Previous findings demonstrated the utility of cytokines/chemokines, such as IFN-γ, IL-2, IP-10, MIG, TNFα, and MCP-1 or CCL2, as biomarkers for monitoring disease recovery [16,17] or treatment efficacy, suggesting that these biomarkers could be indicators of clinical cure in VL individuals [17,18,19,20].

Unlike active VL, the relevance of asymptomatic infection for parasite transmission and disease outcome is largely unknown. Asymptomatic visceral leishmaniasis (AVL) is frequent in endemic areas [21,22], and asymptomatic individuals may have parasites in their blood [23]. A substantial prevalence of *L. infantum* AVL was observed in Operation Iraqi Freedom (OIF) U.S. military deployers; 19.5% had asymptomatic infection defined by a positive cellular/humoral response or nucleic acid testing result [24,25]. AVL presents a concern due to the potential of chronically infected individuals to transmit disease via blood transfusion and organ donation, the possible domestic transmission risk given a permissive and widespread U.S. sand fly vector, *Lutzomyia shannoni*, and the reactivation risk associated with the increasing use of immune modulating treatments and immunosuppression [26,27]. The current parasitological, molecular, and serological methods used to identify asymptomatic infection are resource-intensive and not completely suitable for expanded surveillance, making the identification of these individuals a challenge [19,28].

In this context, new biomarkers capable of identifying AVL are needed to determine people at risk for VL activation and to understand the burden of VL in exposed populations. Evaluating AVL in this cohort of American (from a nonendemic country) military deployers who traveled to VL-endemic Iraq and then returned to the U.S. (in contrast to most published work where individuals remain in endemic areas and can be rechallenged by infected sand flies) can further contribute to the development of clinically useful VL tests and biomarkers.

## 2. Material and Methods

### 2.1. Ethics Statement

This study was conducted according to the guidelines of the Declarations of Helsinki, and the research protocol was approved by the Institutional Review Boards (IRB) of the Uniformed Services University (USU) MED 83-2979, April 2015, Walter Reed National Military Medical Center (WRNMMC) 412208-1, May 2015, and the William Beaumont Army Medical Center (WBAMC) 16–29, which deferred to USU approval. Written informed consent was obtained from all participants involved in the study.

### 2.2. Study Population

Iraq Cohort: 200 OIF-deployed asymptomatic participants completed a leishmaniasis risk factor survey and donated blood samples for VL research assays. Deployed participants were U.S. military personnel in good health, aged 18–60 years, who traveled to Iraq between 2002–2011. Enrollment occurred at WRNMMC/USU in Bethesda MD, DiLorenzo Tricare Health Clinic-Pentagon, Washington, DC, and WBAMC in El Paso TX [25]. From this cohort, 35 participants who tested positive for visceral leishmaniasis and 14 controls (AVL-negative military healthcare beneficiaries) were included in the present study. Asymptomatic VL diagnosis was assessed if any results were positive on testing the peripheral blood samples with quantitative *L. infantum* PCR [29], soluble *Leishmania* Antigen (SLA) ELISA [24], *L. infantum* interferon-γ release assay [24], or rK39 immunochromatographic rapid test (Kalaazar Detect™, InBios, Seattle, WA, USA). The laboratory characteristics of the Iraq AVL cohort have been published [24].

### 2.3. Soluble Leishmania Antigen Preparation

*L. infantum* (MHOM/BC/00/1669, courtesy of Dr. M. Wilson, University of Iowa, Iowa City, IA, USA) parasites were grown at 26 °C in hemoflagellate-modified minimal essential medium with 10% fetal bovine serum until reaching stationary phase [30,31]. SLA was prepared as previously described [32].

### 2.4. Cell Culture

Peripheral blood mononuclear cells (PBMC) were isolated from whole blood as previously described [33]. Briefly, 1 × 10^6^ PBMC were stimulated (or not) with 20 μg/mL SLA for 72 h at 37 °C, 5% CO_2_. Supernatants were collected and stored at −80 °C until used.

### 2.5. Quantification of Biomarkers

The levels of chemokines (RANTES, Eotaxin, MIP-1α, MIP-1β, MCP-1, IP-10, MIG, IL-8) and cytokines (IL-1β, IL-1RA, IL-2, IL-2R, IL-4, IL-5, IL-6, IL-7, IL-10, IL-12, IL-13, IL-15, IL-17, IFN-γ, IFN-α, GM-CSF, TNF-α) in the supernatants of PBMCs from 35 AVL^-^positive (AVL^+^) and 14 control participants stimulated in vitro with soluble *Leishmania* antigen (SLA) for 72 h were assessed by Luminex^®^ technology (Cytokine 25-Plex Human Panel, Invitrogen, Waltham, MA, USA). Data acquisition and cytokine/chemokine levels were measured on a Luminex^®^ 200 System or Bioplex^®^ Manager Software using the Five-Parameter Logistic Regression, with results expressed in pg/mL or baseline fold.

### 2.6. Statistical Analysis

Statistical analysis was carried out using GraphPad Prism v.9.0 Software (San Diego, CA, USA) and Stata 17 SE for the combinatorial ROC curve [34]. Comparative analysis between any two groups was performed using the nonparametric Mann–Whitney U test, according to the data distribution. The differences were considered significant if *p* < 0.05. The performance and cut-off for each analyte were determined by calculating the area under the receiver operating characteristic (ROC) curve (AUC) and the 95% confidence interval (CI).

Levels of chemokine/cytokine were expressed as pg/mL (value for SLA-stimulated culture subtracted by the value for un-stimulated culture from the same person) or baseline fold (value for SLA-stimulated culture divided by the value for un-stimulated culture from the same person). Due to an intrinsic variation of the manufactured kits in the standard curve levels (pg/mL) of some chemokine/cytokine, the fold increase of some analytes was calculated instead of presenting the concentration values. The majority of the time, when individual chemo/cytokine standard curve values correlated from kit to kit, results were provided as analyte concentration.

## 3. Results

### 3.1. Characteristics of U.S. Military Study Volunteers

The mean age of AVL volunteers was 40.7 years; the majority were male and self-reported white race. Demographics of the cohort are presented in Table 1.

### 3.2. IL-8, MCP-1, and MIG produced by SLA-stimulated PBMC Are Useful Biomarkers for the Identification of People with AVL

**Figure 1:**
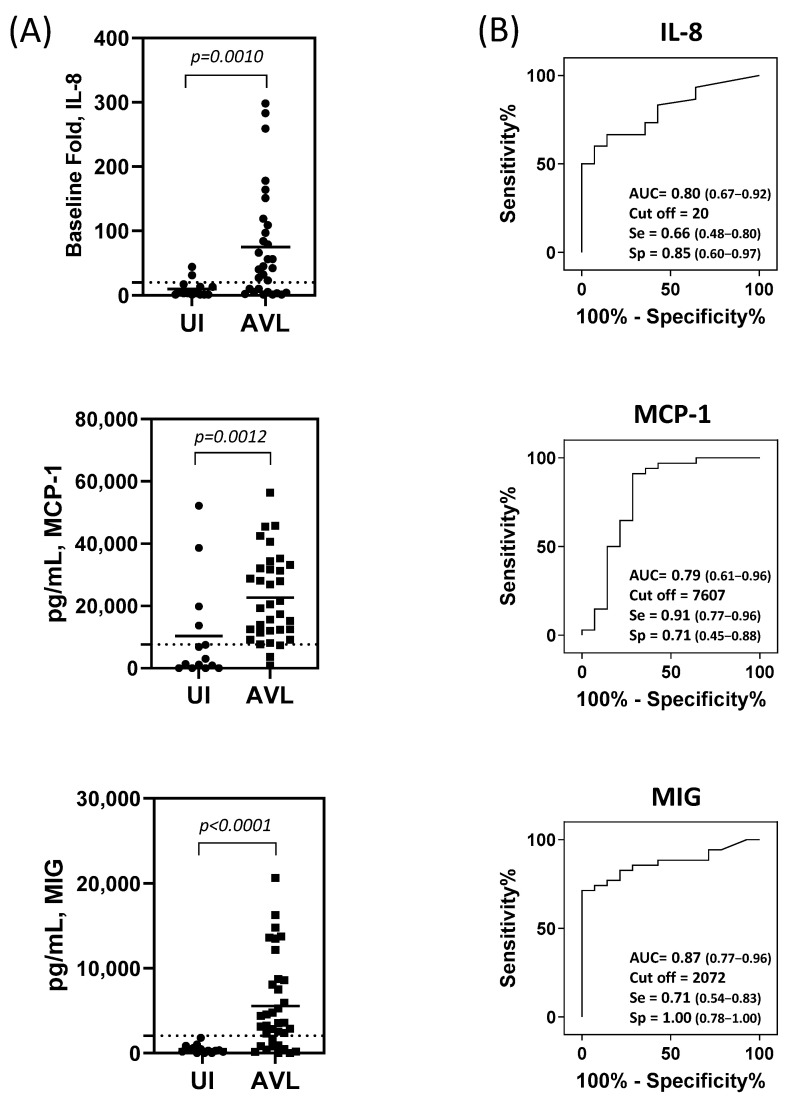
Chemokine production in supernatants of cultured PBMCs from study participants. (**A**) Production of chemokines following 72 h of SLA stimulation of PBMC from individuals with asymptomatic visceral leishmaniasis (AVL^+^) and uninfected (UI, AVL negative) military healthcare beneficiaries. Biomarkers IL-8 (UI, n =14 and AVL, n = 30); MCP-1 (UI, n =14 and AVL, n = 34), and MIG (UI, n =14 and AVL, n = 35). Horizontal bars represent the mean concentration for each biomarker. Dashed lines represent the cut-off. The identification of the cut-point value requires a simultaneous assessment of sensitivity and specificity. A cut-point will be referred to as optimal when the point classifies most of the individuals correctly. (**B**) Receiver operating characteristics curve analyses to determine the performance of these analytes as potential biomarkers for AVL. AUC = area under the curve, se = sensitivity and sp = specificity. Statistical analyses were performed using Mann–Whitney U test.

Cytokine and chemokine levels were analyzed in PBMC supernatants collected from an AVL^+^ Iraq-deployer cohort (Figure 1). Several chemokines/cytokines presented no or low detected levels, with no significant difference from controls (Supplementary Table S1). However, three analytes were found at significantly higher concentrations in the AVL^+^ cohort compared to the AVL negative controls: IL-8 (*p* = 0.0010), MCP-1(*p* = 0.0012), and MIG (*p* < 0.0001) (Figure 1A). Receiver operating characteristic (ROC) curves were built to evaluate the performance of each analyte as a possible biomarker for AVL. Our data demonstrated that MIG was the best biomarker, with an area under the curve (AUC) value of 0.87 and 100% specificity (sp). However, MCP-1 was more sensitive (se) than MIG (91% versus 71%). IL-8 and MCP-1 performed moderately well with AUC = 0.80 (se = 0.66, sp = 0.85) and AUC = 0.79 (se = 0.91, sp = 0.71), respectively (Figure 1B).

### 3.3. Combinatorial Analysis for Identification of Individuals with AVL

**Figure 2:**
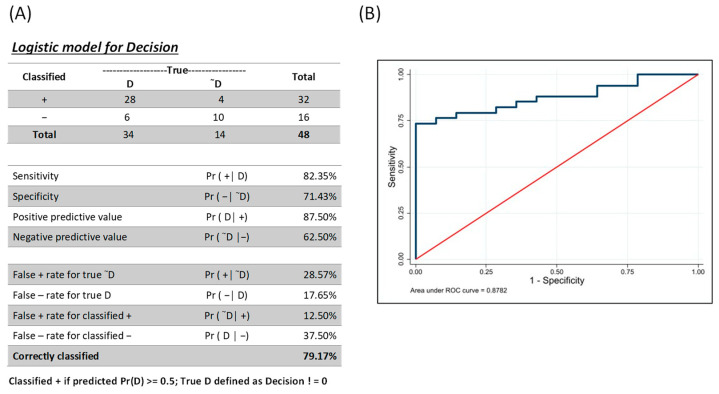
Combinations of MIG and MCP-1 markers identified by combined ROC. (**A**) Logistic model for decision showing the fraction of predictions using a cutoff of predicted probability 0.5 (FN: false negative; FP: false positives; TN: true negative; TP: true positive) and the percentage of correctly classified. (**B**) Receiver operating characteristic curve analysis to determine the performance of these analytes combined as potential biomarkers for AVL. AUC = area under the curve.

To determine whether a combination analysis of the MIG and MCP-1 biomarkers would increase the analyte performance, we generated a combined ROC using the *lroc* function and post-estimation for logistic regression in Stata (generation of the marker combinations based on se and sp filters) as described by [35]. Using a multiple logistic regression where all biomarkers were used as predictors, a resulting predicted probability of 0.5 was considered a case- or disease-positive observation (Figure 2). Using that as a cutoff, the model correctly classified 79.17% of the observations, with a sensitivity and specificity of 82.35% and 71.43%, respectively (Figure 2A). The ROC curve provided (Figure 2B) is a visual representation of all possible combinations of sensitivity and specificity from these inputs, with a possible AUC of 87.82%.

The ROC curve of MIG and MCP-1 markers in combination is presented in Figure 2B. Our results demonstrated that the combinatorial analysis did not improve the overall performance when compared to the MIG performance alone.

## 4. Discussion

The control of *Leishmania* parasites within the host cell is mediated by innate and adaptive immune responses [36]. Following infection, both pro- and anti-inflammatory cytokines and chemokines are secreted, which have dynamic effects in stimulating immune cells to react against *Leishmania* [37,38].

Previous studies showed that following antigen stimulation in vitro, PBMCs of symptomatic patients with VL exhibited a predominantly type 2 response, whereas cells of AVL^+^ individuals produced a mixed cytokine profile [9]. This mixed response ensures both effective control of parasite proliferation and preservation of immune homeostasis by blocking an excessive cellular response [11]. A mixed cytokine pattern (IFN-γ produced by CD8^+^ and CD4^+^ cells and IL-5 produced by CD4^+^) was also associated with asymptomatic *L. infantum* infection [39]. On the other hand, recently, a scoping review (selected 106 articles) debated whether the cellular immunity of asymptomatic VL was associated with a T1-type cellular response, with elevated levels of IFN-γ, TNF, IL-2, IP-10, IP-10 (CXCL10), MIG (CXCL9), MCP-1 (CCL2), neopterin, and soluble CD40 ligand, when low levels of IL-10, IL-4, and IL-17 were detected [40].

In the present work, cytokine/chemokine levels were assessed in a unique AVL^+^ cohort of U.S deployers. Although some chemokines/cytokines (from the large panel assessed) were not detected, the levels of specific chemokines were higher in AVL^+^ participants, especially MIG, MCP-1, and IL-8, suggesting their utility as biomarkers for identifying asymptomatically infected individuals.

Our results demonstrated that Monokine induced by Gamma (MIG) had the best performance when comparing the three identified biomarkers. MIG (or CXCL9) is a chemokine induced by IFN-γ and has the potential to provide amplification of the IFN-γ signal. Ritter and Korber [41] demonstrated the key role of MIG in the self-healing of localized cutaneous leishmaniasis, confirming that lesions exhibited a strong expression of Th1-associated chemokines, such as CCL2/MCP-1, CXCL9/MIG, and CXCL10/IP-10. More recently, it was shown that IL-8, IP-10, and MIG were robust markers for identifying asymptomatic VL from *L. infantum* (Spain) and *L. donovani* (Bangladesh) endemic areas [42]. MIG was reported as 100% sensitive and specific for those with VL [42]. However, previous studies demonstrated that MIG can also be produced at different disease stages (active VL, VL after treatment, asymptomatic) and may not be specific to AVL [43].

Our data suggest that MCP-1 and IL-8 also perform adequately as AVL^+^ biomarkers, although not as well as MIG. MCP-1 can activate anti-*Leishmania* macrophage-killing mechanisms and was studied as a biomarker of cure in cutaneous leishmaniasis [4]. High MCP-1 expression in the skin is described in localized, self-healing cutaneous lesions [44], but no detectable MCP-1 is found in diffuse, non-healing cutaneous lesions [41,45]. Our findings extend those of others, as MCP-1 was identified as a potential biomarker for asymptomatic individuals exposed to *L. infantum* and was proposed to measure VL treatment efficacy [19].

Among our healthy AVL+ cohort, SLA-stimulated mononuclear cells produced elevated IL-8/CXCL8 chemokine levels. Neutrophils are stimulated by IL-8-inducing chemotaxis during systemic inflammation, so IL-8 is broadly used as a biomarker of diverse inflammatory processes. Interleukin-8 also induces granulocyte degranulation [46]. Activation of human keratinocytes in response to *L. infantum* was associated with upregulated transcripts for IL-6, IL8, and CXCL5 that may limit the replication of intracellular parasites [47]. A reduced neutrophil count during active VL is associated with lower IL-8 levels in serum [48]. Tasew et al. [49] observed that in response to *L. donovani* and lipopolysaccharide stimulation, cultured whole blood from those with active VL showed significantly lower secretion of IL-8 and IP-10 compared to endemic healthy controls. Among malnourished individuals with active VL, *L. donovani* stimulation was associated with significantly less IL-8 and MIP-1α production [50]. Polymorphisms at the IL-8-251 position are associated with impaired IL-8 activity and the development of active VL in Iranian individuals [51], but this was not demonstrated in Brazilian patients with VL, where the T allele in position -509 of the TGFB1 gene was associated with both symptomatic and asymptomatic VL compared to *Leishmania* skin test-negative individuals [52]. In Bangladesh, those with symptomatic VL and asymptomatic “subclinical” VL had 14–19% rates of elevated IL-8, while those recently treated or “preclinical”, meaning within one year they developed symptomatic VL, had no IL-8 circulating [53]. Among 40 Spanish *L. infantum* AVL and 12 Bangladesh *L. donovani* AVL, IL-8/CXCL8 was associated with an AUC of 0.96 in stimulated plasma and 0.87 in dried plasma spots, although MIG/CXCL9 and IP-10/CXCL10 performed better [43].

The combined ROC is a user-friendly, reliable analytical tool to support researchers in the selection of optimal marker combination(s) through an interactive interface. It allows users to make choices based on the evaluation of all possible markers, double-filter scoring on sensitivity and specificity, selection of the best-performing combinations, and visualization of their receiver operating characteristic (ROC) curves. Based on that, we decided to evaluate the combination of our best biomarkers; however, for AVL^+^ biomarkers, it does not improve the sensitivity/specificity.

Our study has a few limitations due to including a relatively modest number of participants and measuring the cytokine/chemokine levels at 72 h post-stimulation, thus not accounting for cytokines peaking at earlier timelines (e.g. IL-4 typically secreted at 24 h). Moreover, although described as a powerful tool, the Luminex^®^ platform does not have a high dynamic range, so cytokines secreted at low levels are not usually detected. We discussed in a prior report [24] that there may be some limited cross-reactivity in immune responses to our *Leishmania* antigen between species. In addition, the studied population was assessed after an average of 10 years after deployment to Iraq, so it could potentially be studied when waning immune responses. Despite these limitations, our study was unique as the cohort of participants had limited exposure to *Leishmania* during deployment and no continuous reinfection opportunities (as compared to living in endemic areas).

Depicting the biological implications of asymptomatic infection is critical for planning and evaluating VL control measures in endemic regions as well as developing strategies for the individual with infection. We identified that 64% of this cohort had antibodies to the saliva of the sand fly *Phlebotomus alexandri*, a VL vector in Iraq [25], suggesting that U.S. Forces had high exposure to the vector. Quantifying circulating immunological biomarkers as an alternative strategy for monitoring asymptomatic visceral infections will improve the identification of people at risk of developing VL disease. During a 16-year surveillance period among members of the U.S. Armed Forces, 25 active cases of visceral leishmaniasis were reported, representing 1.2% of the total leishmaniasis cases [54]. Late reactivated visceral leishmaniasis presenting as a lingular ulcer was described in a U.S. service member deployed during Operation Desert Storm in 1991 and Operation Iraqi Freedom (2002–2003) [55]. Therefore, the potential biomarkers described in our study can provide a further method to detect risk for activation of VL among U.S. veterans that is less complex than the multiple diagnostic strategies (serology, IGRA, qPCR) we employed in our surveillance for asymptomatic VL. The persistence of these chemokines, on average nearly a decade after infection in Iraq, raises theoretical concerns for accelerated immunoaging and risk of T cell exhaustion with persistent inflammatory stimuli.

These identified markers, MIG, MCP-1, and IL-8, may be useful to determine the risk of developing overt VL in individuals. Monitoring biomarkers may be used for individual or population-level surveillance, in this case for asymptomatic VL [56]. These biomarkers can be used as susceptibility/risk biomarkers that indicate the potential for developing a disease in a person who does not currently have a clinically apparent disease [57]. U.S. servicemembers are not tested for visceral leishmaniasis in the absence of symptoms outside of research studies, and there are no leishmaniasis restrictions on the donation of blood or other tissues except if VL has been confirmed with validated testing, such as invasive tissue biopsies. To further develop MIG as a biomarker, qualification would require concrete characterization of the biomarker to the outcome of interest, testing large numbers of the millions who deployed and following them over decades to determine who activates VL. Given that our biomarkers require cells, the Department of Defense’s serum repository would not suffice, although perhaps the Million Veterans Program of the Department of Veterans Affairs genomic material collection could be leveraged. Reproducibility and a better understanding of sources of variability in chemokine measurement, values over time in the same individual, analytic validation, and assessing specificity for *Leishmania infantum* would be necessary for this to become a clinically available marker. The applicability for those who deployed in the past and are now starting immunosuppression seem to be a target population at particular risk for activation. The natural history of asymptomatic VL continues to be incompletely studied, something that could provide information to buttress treatment mitigation strategies and understand the context of its use.

In conclusion, our results highlight that MIG is a promising biomarker for the identification of asymptomatic visceral leishmaniasis using peripheral blood samples. Moreover, IL-8 and MCP-1 may be elevated in AVL as well. Thus, understanding immune responses in asymptomatic infections can contribute to the development of new point-of-care tests and biomarkers that could have clinical applications.

## Figures and Tables

**Table 1:** Characteristics of Study Participants.

Population	AVL Negative Controls (n = 14)	AVL^+^ Participants (n = 35)
**Mean age, years** (range)	35.1 (25–57)	40.7 (29–58)
**Sex** Female Male	5 (35.7%) 9 (64.3%)	3 (8.6%) 32 (91.4%)
**Race/Ethnicity**		
African American	2 (14.3%)	5 (14.3%)
White		
Hispanic/Latino	0	5 (14.3%)
Not Hispanic/Latino	9 (64.3%)	22 (62.8%)
Other	3 (21.4%)	3 (8.6%)
**Positive *Leishmania* Assay** IGRA+ ELISA+ qPCR+	negative negative negative	27 (77.2%) 6 (17.1%) 2 (5.7%)

IGRA = IFN-γ release assay; ELISA = enzyme-linked immunosorbent assay; qPCR = quantitative polymerase chain reaction.

## Data Availability

Additional data supporting the reported analyses are available upon request from the corresponding author.

## Supplementary Materials

The following supporting information can be downloaded at: https://www.mdpi.com/article/10.3390/pathogens12050705/s1, Table S1: Chemokine/Cytokine production in supernatants of PBMC culture from participants.
